# Polycyclic Aromatic Hydrocarbons Induced by Smoking and Air Pollution: Correlation with Oxidative Stress in Chronic Obstructive Pulmonary Disease Patients

**DOI:** 10.3390/toxics10110681

**Published:** 2022-11-11

**Authors:** Ioana Buculei, Mona Elisabeta Dobrin, Daniela Matei, Ilie Onu, Cristina Vicol, Ionel Bogdan Cioroiu, Marius Niculaua, Daniel Andrei Iordan, Andrei Cernomaz, Antigona Carmen Trofor

**Affiliations:** 1 Department of Biomedical Sciences, Faculty of Medical Bioengineering, University of Medicine and Pharmacy “Grigore T. Popa”, 700115 Iasi, Romania; 2 Doctoral School, Faculty of Medicine, University of Medicine and Pharmacy “Grigore T. Popa”, 700115 Iasi, Romania; 3 Medical Science Department, University of Medicine and Pharmacy, Iuliu Hațieganu, 400347 Cluj-Napoca, Romania; 4 Clinical Hospital of Pulmonary Diseases, 700115 Iasi, Romania; 5 Doctoral School of the Faculty of Chemical Engineering and Environmental Protection “Cristofor Simionescu”, Technical University “Gheorghe Asachi”, 700050 Iasi, Romania; 6 Romanian Academy, Iași Branch, Research Center for Oenology, 700505 Iasi, Romania; 7 Department of Individual Sports and Kinetotherapy, Faculty of Physical Education and Sport, “Dunărea de Jos” University of Galati, 800008 Galati, Romania; 8 Center of Physical Therapy and Rehabilitation, “Dunărea de Jos” University of Galati, 800008 Galati, Romania; 9 Faculty of Medicine, University of Medicine and Pharmacy “Grigore T. Popa”, 700115 Iasi, Romania

**Keywords:** COPD, oxidative stress, air pollution, PAHs, smoking, uric acid, MDA

## Abstract

Oxidative stress is induced by tobacco smoking and is also associated with exposure to air pollution, which are two of the most important risk factors for chronic obstructive pulmonary disease (COPD). The aim of this study was to correlate tobacco use and exposure to air pollution with oxidative stress markers useful in clinical practice in patients with COPD. A total of 102 patients were included and the levels of polycyclic aromatic hydrocarbons (PAHs), malondialdehyde, uric acid and number of packs-years (PY) were determined. Also, six different ratios were used to assess the source of exposure. The results obtained in this study show an admission of pollutants according to smoking status (former smokers/smoker/non-smokers) quantified in average total concentrations for the group of patients with COPD of 4.12 ng/mL, 6.76 ng/mL, 6.04 ng/mL. The six ratios used show that in COPD, the content of PAHs in the blood could be a result of diesel emissions and fuel combustion. Uric acid levels were lower in the smoker group of COPD patients (mean = 5.21 mg/dL), which indicates that oxidative stress is intensified with each cigarette smoked. Additionally, high concentrations of malondialdehyde were quantified for smoking patients diagnosed with COPD (mean = 2.72 µmol/L) compared to former smokers (mean = 2.43 µmol/L) and non-smoking (mean = 2.32 µmol/L) patients, which is another indicator of the implication of smoking in oxidative stress in COPD patients.

## 1. Introduction

According to the 2019 global report of the World Health Organization (WHO), currently, chronic obstructive pulmonary disease (COPD) is the third leading cause of death worldwide, being responsible for 3.23 million deaths in 2019, with 80% of these deaths occurring in countries with low and medium incomes [1]. The Global Initiative for Chronic Obstructive Lung Disease (GOLD) 2022 defines COPD as a common, preventable and treatable disease characterized by persistent respiratory symptoms and airflow limitations due to airway and/or alveolar abnormalities usually caused by significant exposure to noxious particles and gases [2].

The changes that occur in the respiratory system of a COPD patient are mainly due to chronic inflammation caused by exposure to tobacco smoke, and also by exposure to other harmful particles. Smoking is recognized as the main risk factor for the development of COPD. However, studies have shown that half of smokers who are highly addicted to nicotine do not develop COPD, and that non-smokers may also develop COPD throughout their lives. Despite reaching peak lung function in early adulthood, some people may develop COPD by exposure to and inhalation of harmful particles from the atmosphere that cause an accelerated decline in lung function [3].

Polycyclic aromatic hydrocarbons (PAHs) are a class of complex organic substances, consisting of carbon and hydrogen atoms that contain at least two benzene rings [4]. PAHs are found everywhere in the environment, respectively in air, water and soil (including sediments), because they use the atmosphere as means of dispensation. They can be found as such in tar sediments and coal, but can also be generated by the incomplete combustion of organic matter, for example, forest fires, incineration and engines. It is known that PAH compounds such as fluoranthene, benzopyrene, anthracene and acenaphthylene are capable of producing toxic, mutagenic and carcinogenic effects, being an important risk factor for respiratory diseases, such as lung cancer [5].

There is evidence that at the level of the respiratory system of COPD patients, an imbalance between oxidants and antioxidants concentration is present, which causes oxidative stress. Oxidative stress reflects an imbalance between the presence of oxidants and the ability of a biological system to detoxify these reactive oxidants or repair the damage they cause [6]. Both exposure to tobacco smoke and exposure to air pollutants produce oxidative stress at the respiratory system level. Oxidative stress plays an important role in the onset and maintenance of inflammation in the respiratory tract. Researchers have shown that smokers have high levels of oxidative stress, and exposure to PM_2.5_ causes oxidative stress and inflammation in healthy mice prior to exposure [7].

In patients with COPD, oxidative stress can be the result of reactive oxygen species (ROS) present in cigarette smoke and/or may be caused by inflammatory and immune stimuli present in the respiratory epithelium. At the same time, oxidative stress can increase the intensity of lung inflammation by recruiting and activating immune cells in the lungs, and thus increasing the production of inflammatory mediators. For this reason, it is considered that the antioxidant capacity of patients is very important and dictates the severity and progression of COPD [6,8]. Oxidative stress can also be induced by exposure to PAHs. Toxicity at the cellular level is imposed by the electrophilic intermediates, resulting from the metabolic activation of these compounds, caused by their capacity for covalent binding to DNA or by their capacity for engaging in the redox cycle. The result will be an over-production of reactive oxygen species (ROS), which in turn causes oxidative stress [9].

The body must protect itself from the effects of oxidative stress and does so with the help of multiple interdependent enzymatic and non-enzymatic antioxidants. Non-enzymatic antioxidants include uric acid (UA), metal-binding proteins (MBPs), bilirubin (BIL), glutathione (GSH), melatonin (MEL) and polyamines (PAs) [10,11]. Uric acid is considered one of the most important low-molecular-mass antioxidants of the human body because of its radical scavenger proprieties of peroxyl radical, hydroxyl radicals and singlet oxygen; also, uric acid can chelate metal ions and convert them to poorly reactive forms that are incapable of catalyzing free-radical reactions [12]. A study conducted by Haj Mouhamed et al. has shown that smoking may cause uric acid levels to drop secondary to a reduction in the endogenous production caused by high levels of oxidative stress [13].

The leading source of oxidative damage is represented by lipid peroxidation, which is a direct effect of oxidative stress. Oxygen radicals oxidize polyunsaturated fatty acid-producing lipid hydroperoxides and aldehydes. The resulting products are used as biomarkers of oxidative stress. Malondialdehyde (MDA) is the biomarker most often used to assess oxidative stress levels in patients with COPD and other diseases [14].

The aim of this study was to investigate the impact that cigarette smoking and exposure to air pollution, measured as serum levels of PAHs, have on the oxidative stress level of patients diagnosed with COPD compared with patients with other respiratory diseases. The antioxidant response was assessed by the quantification of serum uric acid level, and malondialdehyde was used as a biomarker of oxidative stress. To the best of our knowledge, this is the first study conducted in Romania that investigates the role of smoking and exposure to air pollution in inducing oxidative stress in COPD patients.

## 2. Materials and Methods

### 2.1. Study Group

A total of 102 subjects from the Clinical Hospital of Pulmonary Diseases, Iasi, Romania were included in this study. This hospital is a reference center for the treatment of pulmonary diseases in the north-east region of Romania. Informed consent was obtained from every participant in the study. The patients included in the study were divided into two groups; one group was formed of patients diagnosed with different stages of COPD (COPD group), and the other group contained patients diagnosed with other respiratory diseases (Control group). Blood samples were collected in the morning after 8 h of overnight fasting and were provided from the biochemistry laboratory of the hospital; biochemistry analyses were performed and blood serum samples were preserved and stored at −25 °C. Smoking habits (number of cigarettes smoked per day, years of smoking), geographical location of residences, type of domestic heating, and possible exposure through occupational and non-occupational factors were detailed in an exhaustive inquiry form. In addition, questions about age, gender and secondary diagnostics were included in the questionnaire.

### 2.2. Ethical Considerations

The study was approved by the ethics committee of the Clinical Hospital of Pulmonary Diseases, Iasi, Romania (ethical approval no. 83/22.04.2021) and by the ethics committee of the University of Medicine and Pharmacy “Grigore T. Popa” Iasi, Romania (ethical approval no. 9205/09.06.2020). All participants offered their written informed consent to participate in the study and for their extracted medical data to be used in accordance with the GDPR regulations. The rules related to the deontology of the academic writing of scientific articles and the processing of personal data were respected [15]. 

### 2.3. Quantification of PAHs

#### 2.3.1. Standards and Reagents

Mixtures of 16 PAHs, naphthalene (N), acenaphtene (Ace), acenaphthylene (Acy), fluorene (Fl), phenanthrene (P), anthracene (A), fluoranthrene (Flu), pyrene (Py), benzo(a)anthracene (BaA), benzo(b)fluoranthrene (BbFlu), benzo(k)fluoranthrene (BkFlu), benzo(a)pyrene (BaPy), dibenzo(a,h)anthracene (DahA), benzo(g,h,i)perylene (BghiPy), chrysen (Ch) and indeno1,2,3-cd-pyren (IPy), were purchased from Sigma Aldrich.

A mixture of sixteen PAHs (naphthalene, acenaphthene, acenaphthylene, fluorene, phenanthrene, anthracene, fluoranthene, pyrene, benzo(a)anthracene, benzo(b)fluoranthene, benzo(k)fluoranthene, benzo(a)pyrene, dibenzo(a,h)anthracene, benzo(g,h,i)perylene, chrysene, indeno1,2,3-cd-pyren) was used as the starting stock standard solution and phenanthrene D10 as internal standard and these were obtained from Dr. Ehrenstorfer—LGC Standards (Manchester, NH, USA). The PAHs mixture was dissolved in acetonitrile, the quantity of each component in the mixture was 10 μg/mL each, and their purity was of 99.99%. Working standards and also internal standards for the calibration curve were obtained from the successive dilution of the starting stock standard solutions. Methanol, acetonitrile, dichloromethane, and water were of HPLC purity and were purchased from Merck—Darmstadt, Germany. The other types of working materials involved in the sample preparation process were C18 cartridges of Mega Bond Elute Plexa from Agilent Technologies USA (Santa Clara, CA, USA) with a column bed of 500 mg and volume of 6 mL. Solid phase extraction was performed on Supelco Visiprep SPE vacuum system.

#### 2.3.2. Instrumentation

Chromatographic determinations were performed on a high-performance liquid chromatograph Nexera X2—Shimadzu, Japan with Lab-Solutions software, equipped with a LC-30AD pump, SIL-30AC autosampler, column thermostat CTO-30A and a fluorescence detector RF-10AXL with a high-energy xenon lamp. Analyses were performed on a chromatographic column Pinnacle DB PAH Restek USA (Bellefonte, PA, USA) with 100 mm length, 2.1 mm internal diameter and 1.9 μm particle size.

#### 2.3.3. Chromatographic Method

The parameters of the chromatographic method were derived from another study [16], with minor modifications. The mobile phase consisted of a mixture of water and acetonitrile and the chromatographic elution used a gradient at flow rate of 0.54 mL/min starting with 50% acetonitrile and 50% water for 8 min, then the acetonitrile component increased up to 90% for 6 min and was maintained constant up to 18.5 min. The column temperature was 30 °C. Excitation and emission wavelengths specific to every category of PAHs were settled according to a previously mentioned study. Acenaphthylene (Acy) has not been quantified because it has no native fluorescence.

#### 2.3.4. Standard Preparation

For the preparation of samples used in calibration, a series of volumes of PAH starting stock standard solution were used to produce a series of concentrations of 100, 75, 50, 25, 10 and 5.0 ng/mL of each component. For every solution a concentration of 100 ng/mL internal standard was used.

#### 2.3.5. Sample Preparation

A volume of 1.0 mL human serum (plasma) was mixed with 0.1 mL internal standard stock solution (250 ng/mL) and diluted to 20 mL with water. SPE cartridges were conditioned with 6 mL dichloromethane and 6 mL methanol followed by 6 mL water. Then the samples were loaded on the SPE cartridge, followed by column washing with 12 mL water. The next step was column drying for 30 min at high vacuum. The elution of components was performed with 5 mL dichloromethane. The eluate was evaporated under nitrogen and the residue was reconstituted with 0.25 mL acetonitrile. A volume of 5 μL was injected into the column.

### 2.4. Quantification of MDA

#### 2.4.1. Standards and Reagents

The MDA was of analytical standard, 99.9%, and was procured from Sigma Aldrich—Burlington, MA, USA. 3-nitrophenylhydrazine hydrochloride (3NPH*HCl) was procured from TCI Chemicals Japan (Nasukarasuyama, Japan). Trichloroacetic acid (TCA), trifluoroacetic acid (TFA), sodium hydroxide and formic acid were of analytical purity. Water was produced from a Merck Millipore system and acetonitrile of HPLC purity was supplied by Merck KGaA—Darmstadt, Germany. As internal standard, ^13^C_6_-3 nitrophenyl-1-pirazole (^13^C_6_-3NPH*HCl) was used and this was procured from Cayman Chemicals (Ann Arbor, MI, USA).

#### 2.4.2. Instrumentation

Analyses were performed on a Thermo Scientific (Waltham, MA, USA) system Transcend TLX equipped with binary pump HPG-3200 RS, column thermostate TCC-3000RS, valve interface module and an open auto-sampler PAL-LX1. The chromatographic system was coupled with a TSQ Quantum Access Max triplequadrupol mass spectrometer, with HESI ionization source. Chromatographic separation was performed on a Zorbax Eclipse Plus C18 column (50 mm length, 4.6 mm internal diameter and 1.8 μM particle size). The mobile phase and mass spectrometer conditions were the same with slight modifications in terms of optimization as those from the work of Sobsey et al. [17].

#### 2.4.3. Standard Preparation

For derivatization, 3NPH*HCl was used at a concentration of 100 mM by dissolving 1.896 g of 3NPH*HCl in a mixture of 75% acetonitrile with 0.2% TFA in water and diluted to 100 mL with the same solvent.

Internal standard preparation was performed using ^13^C_6-_3 nitrophenyl-1-pyrazole at a concentration of 10 μM by adding 200 μL of 1.25 mM MDA solution in 75% ACN-0.2%TFA to a vial containing 1 mg of ^13^C_6_-3NPH*HCl. The mixture was kept at 50 °C for 30 min then transferred to a 25 mL vial. The mixture was made up to volume with 20% acetonitrile in water, resulting in a concentration of 10 μM ^13^C6-3 nitrophenyl-1-pyrazole.

A series of concentrations of MDA of 3.0, 15, 30, 50, 70 and 100 μM in water was prepared via previous derivatization. The reaction was done by mixing a volume of 50 μL solution with 100 μL 3NPH*HCl and 100 μL water. The mixtures were kept at a temperature of 60 °C for 15 min, then a volume of 50 μL was mixed with 50 μL internal standard solution resulting in a series of concentrations of 0.3, 1.5, 3.0, 5.0, 7.0 and 10 μM.

#### 2.4.4. Sample Preparation

A volume of 50 μL human plasma was mixed with 50 μL water and 25 μL 6M sodium hydroxide solution in a 2 mL Eppendorf test tube. The mixture was vortexed for 6 min and kept at a temperature of 60 °C for 30 min. The samples were acidified with 250 μL solution 20% trichlorcetic acid and centrifuged for 5 min at 18,000 rpm. A volume of 50 μL from a previous solution was mixed with 100 μL 3NPH and maintained at 50–60 °C for 15 min. Finally, a volume of 50 μL was mixed with 50 μL internal standard and injected into LC-MS according to the analytical method.

### 2.5. Biochemical Assay

Serum parameters were measured using a Cobas Integra 400 plus (Roche, Basel, Switzerland) biochemical auto analyzer. All tests were performed at the Biochemical Laboratory of the Clinical Hospital of Pulmonary Diseases, Iasi, Romania, following standard procedures for clinical biochemistry purposes. The biological markers measured were blood serum uric acid, total cholesterol, triglycerides, low density lipoprotein cholesterol (LDL-C) and high density lipoprotein cholesterol (HDL-C).

### 2.6. Quality Assurance

1. *Quality assurance for the PAHs* comprised in injecting spike solutions of 25 ng/mL, 50 ng/mL or 100 ng/mL in all 25 of the samples included in the study. The spiked samples were prepared by using a suitable amount of biological fluid, enriched with a suitable amount of PAHs stock solution and prepared using the solid phase extraction procedure. Recovery was calculated after every sequence in order to verify the stability of the method. In this way, for every spike sample, the average values of the concentrations in each compound were between 80 and 112%. For LOD and LOQ the calculation method is based on the standard deviation of the response (Sy) and the slope of the calibration curve (S). The standard deviation of the response was determined based on the standard deviation of y-intercepts of regression lines. In this way LOQ and LOD were calculated using the formulas LOQ = 10(Sy/S) and LOD = 3.3(Sy/S). The range of LOQ was between 0.01 ng/mL for anthracene and 1.35 ng/mL for phenanthrene. The values of LOD were between 0.003 ng/mL and 0.45 ng/mL for anthracene and chrysene, respectively.
2. *For quality control in the case of the determination of the total form of MDA*, a series of concentrations of 2, 3, 6 and 8 μmol were prepared according to the sample preparation for total malondialdehyde. The samples were prepared by diluting a series of stock solutions of 0.4, 0.7, 1.5 and 2.0 μmol using a dilution factor of 200. The average recovery rate for the 3 concentrations was between 89.15% for 6 μmol and 108.49 for 8.0 μmol samples. The overall coefficient of variation was 6.14%.
3. *Quality assurance in the case of biochemistry markers* was performed using internal quality control and external quality control. Internal quality control was run with lyophilized human serum Cobas Precicontrol ClinChem Multi1 and Precicontrol ClinChem Multi2. The results were interpreted by the standard Westgard rules. In the case of external quality control, the results are interpreted by z scores and standard deviation. In all investigated parameters the z score was <2.

### 2.7. Statistical Analysis

All statistical results were obtained using the STATISTICA 10 package (StatSoft Inc., Tulsa, OK, USA). The normality of the results was tested using the Kolmogorov–Smirnov test. The Spearman correlation coefficient was determined with the aim of measuring the correlations between PAH levels in the blood samples for the control group and the COPD group. A *p*-value < 0.05 was considered statistically significant. The characteristics of the two groups investigated were compared using the Mann–Whitney U test. Descriptive statistical analysis was performed using the EXCEL program and for each biochemical parameter determined in the former smokers, smokers and non-smokers groups the average, standard deviation, median and range were measured.

## 3. Results and Discussion

### Statistical Results

In this observational study, serum PAH levels were assessed in patients diagnosed with COPD at various stages of the disease. A number of 15 PAHs were investigated for 102 patients diagnosed with COPD and other respiratory diseases, hospitalized at the Clinical Hospital of Pulmonary Diseases, Iasi, Romania. The study mainly focuses on the quantification of PAHs in the blood of patients diagnosed with COPD and the assessment of the admission of these contaminants according to smoking status and exposure to air pollution. The quantification of oxidative stress in smokers/non-smokers/former smokers was evaluated based on PAHs concentrations, malondialdehyde and serum uric acid levels for COPD patients. The clinical and demographic characteristics of the investigated patients are presented in Table 1. The COPD group patients had an average age of 59.48 ± 10.96, and 92% of the patients were male and 8% female. From the total patients investigated, 49% were from rural areas and 51% were from urban areas, and following the interview, some stated an occupational exposure (16%) or a passive exposure to tobacco smoke (10%) (Table 1).

The profiles of the levels of PAHs determined in the blood samples for the control group and the group of patients with COPD are presented in Table 2 and Table 3. Bellow can be found the names and abbreviations of the 16 PAHs determined in the present study.

The International Agency for Research on Cancer, 1986, classified PAHs into carcinogenic and non-carcinogenic compounds. Six PAHs have been classified as possible carcinogenic compounds to humans: BaA, B(b), Flu, B(k)Flu, BaPy, Db(a, h)A and IPy. The results obtained in this study show an admission of pollutants according to smoking status (former smokers/smoker/non-smokers) quantified in average total concentrations for the group of patients with COPDs of 4.12 ng/mL, 6.76 ng/mL, and 6.04 ng/mL (Table 2). In the case of the control group, the admission of carcinogenic PAHs was determined at mean concentrations of 1.20 ng/mL, 1.67 ng/mL and 1.99 ng/mL for former smokers/smokers/non-smokers, and these values were much smaller than in the case of the COPD group patients. Similar results for levels of PAHs in the blood have been reported by Neal et al. [18].

**Figure 1:**
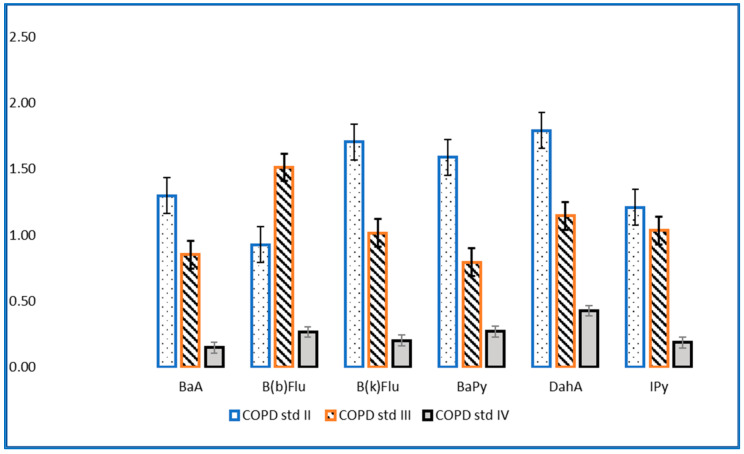
Levels of carcinogenic PAHs (ng/mL) in patients with stage II, III and IV COPD.

**Figure 2:**
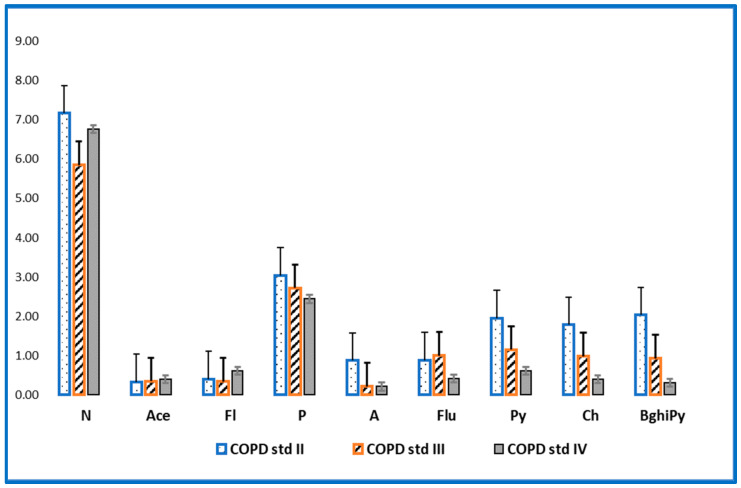
Levels of non-cancerous PAHs (ng/mL) in patients with stage II, III and IV COPD.

The evaluation of PAHs in the investigated samples revealed high concentrations for patients with COPD stage II and III compared to the values of the concentrations in patients with stage IV (Figure 1 and Figure 2). These findings can be explained by the fact that patients with stage IV COPD, due to the extremely decreased lung function and the need for supplemental oxygen, have a very low physical activity capacity, which is reflected in an inability to leave the house, thus being much less exposed to outdoor pollution [19,20]. Lung function can also be affected/diminished by dominant involvement in forms of passive leisure, an aspect that generates a sedentary lifestyle [21]. Physical sports and active leisure activities have a beneficial role in optimizing or improving respiratory function, as a compensation mechanism for problems generated by various vices (smoking, alcohol, drugs, etc.) [22]. Globally, it has been found that young people are doing less and less movement in an organized or freeway [23]. The human being is a whole, and the human body is an organism, not a mechanism, so the synergic concept is required in our mode of understanding [24].

The statistical data show a high Spearman correlation between PAHs in the blood serum of patients with COPD. Significant positive correlations were observed between compounds with four (BaA, r = 0.83, *p* < 0.005), five (BaPy, r = 0.83, *p* < 0.005) and six rings (BghiPy, r = 0.98, *p* < 0.005) in smoker patients (Table 4). In the case of non-smoker patients with COPD, important correlations were observed between compounds with four and five rings (r > 0.98, *p* < 0.005) (Table 5).

For the group of patients diagnosed with COPD, the most common non-cancerous PAHs were naphthalene (mean concentration = 7.27 ng/mL) and phenanthrene (mean concentration = 2.79 ng/mL) and these were determined in almost all samples analyzed (frequency = 90–100%). Some two-ringed PAHs, such as naphthalene, detected at relatively high concentrations in the present study have also been found at high levels in environmental samples collected in the same area [25].

At the same time, it can be easily observed that the levels of non-cancerous PAHs are higher in non-smokers and former smokers than in smokers, and the order of admission is PAHs non-smoker > PAHs former smoker > PAHs smokers. These results imply a double exposure of patients to PAHs—exposure to tobacco smoke but also to air pollution [26].

In the case of carcinogenic PAHs, the most abundant compound was DahA, with a mean concentration of 1.79 ng/mL for the non-smoking group. DahA has been detected in compost, wood preservative sludge, gasoline (high octane number), exhaust condensate of gasoline engines, airborne coal tar emissions, and in airborne coke oven emissions [27,28]. The potency values for DahA are known to vary with the route of exposure [29], and DahA has been reported to be up to 10 times more potent than BaPy [30].

BaPy was significantly higher in the group of smokers diagnosed with COPD (mean concentration = 1.21 ng/mL) and was higher than in the group of former smokers (mean concentration of 0.56 ng/mL), but also compared to the group of non-smoker patients (mean concentration = 0.87 ng/mL). BaPy is one of the most investigated PAHs in both biological and environmental samples [31].

Thus, the profile of carcinogenic PAHs concentrations shows high concentrations in smokers (ΣPAHs = 6.76 ng/mL) compared to non-smokers and ex-smokers, and the order of accumulation is ΣPAHs carcinogenic smokers > ΣPAHs carcinogenic non-smokers > ΣPAHs carcinogenic former smokers [32]. Most of the time, identifying the sources of exposure to pollutants remains the most important step in research studies. Thus, the evaluation of some statistical correlations between compounds (Table 4 and Table 5—Spearman correlation), applications of factorial analysis to identify possible exposure factors, or even the calculation of molecular ratios are attempted.

**Figure 3:**
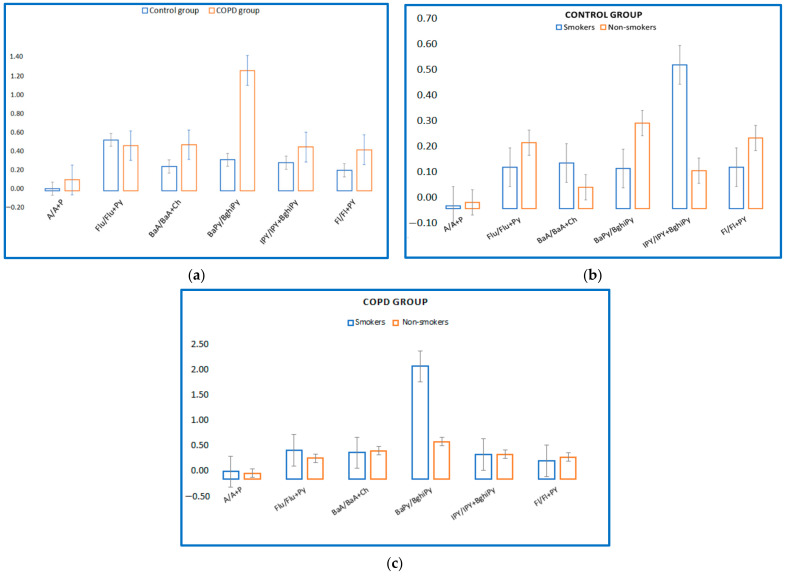
(**a**) Levels of molecular PAHs in blood serum of COPD/control group. (**b**) Levels of molecular PAHs in blood serum of control group, smokers/non-smokers. (**c**) Levels of molecular PAHs in blood serum of COPD smokers/non-smokers.

Regarding serum PAHs intake, six different ratios have been proposed that could suggest the source of exposure. In the present study, molecular ratios were investigated for the control group and COPD group (A/A + P; Flu/Flu + Py; BaA/BaA + Py; BaPy/BghiPy and IPy/IPy + BghiP, Fl/Fl + Py proposed by KG Koukoulakis et al. (2020)) to identify the main sources of exposure to PAHs. Figure 3a shows levels of these ratios for the control group and the COPD group. It is thus observed that in the case of the COPD group, the content of PAHs in the blood could be due to PAHs emissions because the ratios Fl/Fl + Py suggest diesel emissions, and the ratios BaA/BaA + Ch and IPy/IPy + BghiPy are derived from vehicle emissions and fuel combustion [33]. The BaPy/BghiPy ratio indicates non-traffic emissions, and it is observed that in the present study the values are much higher in the group of patients with COPD versus the control group, and this could suggest smoking as the main source of exposure. In Figure 3 it can be observed that smokers in the control group had a high ratio of IPY/IPY + BghiPy (Figure 3b), and in the COPD group, smokers had a high ratio of BaPy/BghiPy (Figure 3c).

There are 16 US EPA PAHs classified as priority pollutants with toxicity and high levels of exposure to humans, 8 of which are considered possible human carcinogens, which include BaA, BaPy, B(b)Flu, B(k)Flu, Ch, DahA and IPy [34,35]. Additionally, PAHs are classified in group 2A (probably carcinogenic) or 2B (possibly carcinogenic). In the present study, PAHs from group 2A (BaA, BaPy, DahA) were quantified, and slightly increased concentrations were observed in smoking patients. For the compounds found in group 2B (B(b)Flu, B(k)Flu, IPy) higher concentrations were observed in the group of patients with COPD. The International Agency for Research on Cancer (IARC) considers exposure to PAHs to be the major risk factor for the development of lung cancer, as well as other pulmonary diseases [36].

The factorial analysis applied to the dataset of patients with COPD highlighted three factors that significantly contribute to the separation of the dataset. Thus, with a variance of 52%, the first factor was mainly represented by PAHs (Py, BaA, Ch, DahA, B(b,k)Flu, Ipy) originating from fuel combustion and traffic emissions, but also non-traffic BghiPy and BaPy. The second factor accounts for 13% of the total variation in the dataset and is represented by FL and A, and the third factor is represented by phenanthrene and accounts for 9% of the variation in the dataset. Identifying the factors that lead to the admission of PAHs in the body is difficult to achieve, even if in the present research the content of non-carcinogenic PAHs predominates, and they could be the source of traffic emissions. At the same time, there is a big difference between the concentrations of carcinogenic PAHs between the COPD group and the control group (Table 2), a difference that could be due to exposure to tobacco smoke.PAHs and nicotine induce the production of oxidative stress markers and reduce the number of antioxidants, contributing greatly to the production of oxidative stress due to cigarette smoke [37,38,39].

In the present study, the quantification of oxidative stress was analyzed by assessing PAHs concentrations, MDA and serum uric acid levels in smokers and non-smokers diagnosed with COPD. Table 6 shows mean, median, stdev and range characteristics of serum MDA, uric acid values and lipid profile parameters of smokers/non-smokers/former smokers diagnosed with COPD.

The results in Table 6 show serum uric acid levels in former smokers/smokers/non-smokers patients diagnosed with COPD at different stages of the disease. The descriptive statistics show, in the case of smoking patients, values of the average uric acid concentration of 5.21 mg/dL, results that are much lower compared to the group of non-smoking patients (6.50 mg/dL) but also compared to the group of former smokers (6.09 mg/dL). Similar results have been reported in other studies [13,40], where a reduction in serum uric acid in smokers indicates that oxidative stress is intensified with each cigarette smoked.

Uric acid is the end product of purine metabolism, and is a non-enzymatic antioxidant with an important role in assessing oxidative stress in the body [12]. Lifestyle (diet, age, smoking, alcohol consumption, exercise) and genetic factors may influence serum uric acid levels, as these factors are directly associated with pathophysiology and oxidative stress. Increased oxidative stress can lead to the depletion of antioxidants, including uric acid, and increased oxidants levels.

In the present study, high concentrations of malondialdehyde were quantified for smoking patients diagnosed with COPD (2.72 µmol/L) compared to former smokers (2.43 µmol/L) and non-smoking patients (2.32 µmol/L) (Table 6). Increased lipid peroxidation in the peripheral blood may indicate a systemic increase in oxidative stress largely characterized by the deficiency of the antioxidant system assessed in this study by determining uric acid concentrations. In fact, cigarette smoke, due to the large number of chemicals resulting from combustion (4700 substances, including polycyclic aromatic hydrocarbons, heavy metals, NO) leads to the formation of strong chemical oxidative stress involving virtually all compounds in tobacco, leading to the formation of free radicals [41]. Several previous studies have demonstrated that PAH exposure may induce oxidative stress [32].

In the case of the evaluation of the lipid profile, significant differences were observed for the levels of serum triglycerides in smokers (226 mg/dL) compared to non-smokers (111.91 mg/dL) and former smokers (103.9 mg/dL). Similar values for the level of serum triglycerides in the case of smoking subjects were also obtained in other studies [42], which tried to explain the physiological consequences of exposure to tobacco smoke.

The statistical difference between the investigated groups was tested with the Mann–Whitney U test. Thus, the statistical results show a statistically significant difference (*p* < 0.05) between the groups of patients with stage II COPD and stage IV COPD for the following investigated parameters: P (*p* = 0.02), BaA (*p* = 0.04), BaPy (*p* = 0.03) and uric acid (*p* = 0.0003) (Table 7).

**Figure 4:**
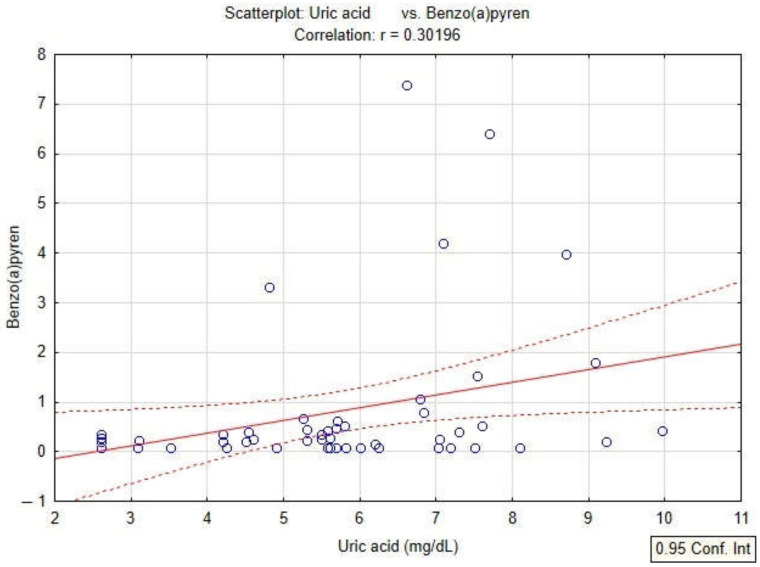
Linear regression between serum uric acid and levels of BaPy in patients with COPD.

**Figure 5:**
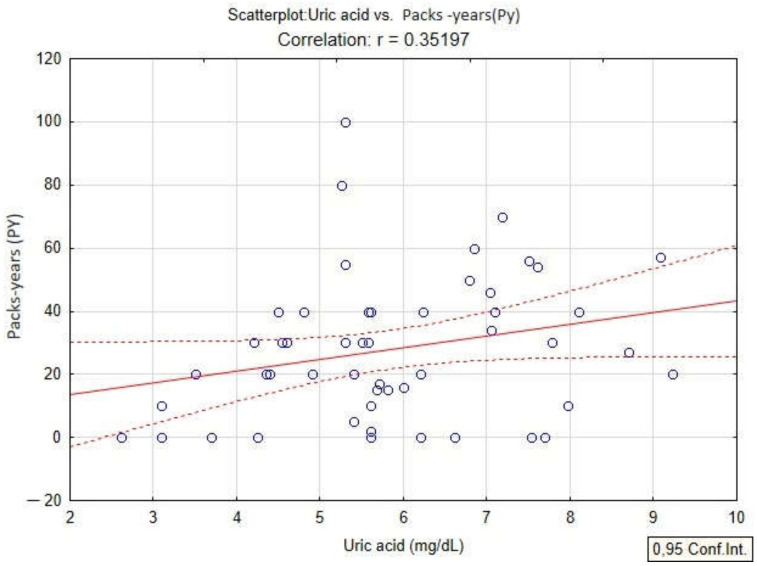
Linear regression between serum uric acid and packs-years (PY) in patients with COPD.

In the present study, the quantification of oxidative stress was studied by analyzing the content of PAHs, serum uric acid values and PAHs in patients diagnosed with COPD. The linear regression model applied to the dataset shows a positive correlation of serum uric acid with BaPy levels (*p* < 0.05, r = 0.30) in smokers and non-smokers diagnosed with COPD, and also with the smoking status of the patients by association with PY (packs-years) (*p* < 0.05, r = 0.35) in smokers diagnosed with COPD (Figure 4 and Figure 5).

The pathogenetic mechanism of oxidative stress caused by exposure to tobacco smoke has not been fully elucidated. A basic hypothesis concludes that free radicals produced by smoking induce oxidative disorders at the macromolecular level (lipids, proteins and DNA). Thus, the results presented in Table 6 quantify high serum levels for triglyceride and low serum levels for HDL-C in smokers (mean triglyceride concentration = 226.99 mg/dL, HDL = 44.9) compared to the group of non-smoker patients (triglycerides = 111, 91 mg/dL, HDL-C = 48.08 mg/dL). Smoking is associated with an atherogenic lipid profile, which can also contribute to the production of oxidative stress. Smoking, in its various forms, leads to an increased risk of high total cholesterol serum levels, as well as high triglycerides levels [40].

## 4. Conclusions

The present study is the first study conducted in Romania that assesses the serum concentrations of PAHs in COPD patients in relation to smoking status and oxidative stress parameters. The results of the study show a high level of PAHs; it was observed that the concentrations of non-carcinogenic PAHs were much higher than carcinogenic PAHs. The most abundant carcinogenic PAHs were BaPy in smokers, DahA for non-smokers and B(b)Flu for former smokers. Six different ratios were used to assess the source of exposure and the results show that in the case of the COPD group, the content of PAHs in the blood could be due to PAHs emissions, because the ratios Fl/Fl + Py suggest diesel emissions, and the ratios BaA/BaA + Ch and IPy/IPy + BghiPy are derived from vehicle emissions and fuel combustion. The statistical results show a positive correlation of serum uric acid levels with BaPy in smoking patients, which could explain the production of chemical oxidative stress by decreasing antioxidant concentrations due to exposure to tobacco smoke. High concentrations of malondialdehyde were quantified for smoking patients diagnosed with COPD compared to former smoker and non-smoker patients, which is another indicator of the implication of smoking in oxidative stress in COPD patients. More studies in this field need to be conducted in order to assess the correlation between exposure to PAHs and the source of exposure and oxidative stress.

## Figures and Tables

**Table 1:** Clinical and demographic characteristics of patients diagnosed with COPD.

Patients Characteristics	BPOC Group (*n* = 51)	Control Group (*n* = 51)
**Variables**		
Age (years)	59.48 ± 10.96	55.01 ± 14.39
Height (cm)	171 ± 0.05	168.6 ± 0.07
Weight (kg)	67.2 ± 8.92	65.3 ± 9.67
Gender		
Male	47 (92%)	32 (63%)
Female	4 (8%)	19 (37%)
Area of residence		
Rural	25 (49%)	24 (47%)
Urban	26 (51%)	27 (53%)
Smoking status		
Former smokers	29 (57%)	3
Non-smokers	9 (18%)	29
Smokers	13 (25%)	19
Packs-years (PY)	34 ± 19.97	22.61 ± 10.68
Primary diagnostic	COPD	Tuberculosis (*n* = 15)
COPD stage I	2 (4%)	Bronchial asthma (*n* = 4)
COPD stage II	12 (24%)	Bronchitis (*n* = 3)
COPD stage III	22 (43%)	Pneumonia (*n* = 7)
COPD stage IV	15 (29%)	Other diseases (*n* = 22)
Occupational exposure to PAHs	16%	1%
Passive smoking	10%	8%

**Table 2:** PAHs concentrations (ng/mL) in human blood serum of patients selected for the control group.

Compound (ng/mL)	(*n* = 51)	Former Smokers				Smokers				Non-Smokers			
Control Group	Frequency (%)	Mean	Median	Stdev	Range	Mean	Median	Stdev	Range	Mean	Median	Stdev	Range
**Non-carcinogenic PAHs**													
**N**	90	9.25	9.44	1.68	9.63–10.7	9.26	9.05	6.70	0–25.65	9.06	8.90	6.40	0–25.9
**Ace**	16	0.05	0.02	0.08	0–0.14	0.13	0.00	0.21	0–0.21	0.17	0.11	0.58	0–2.70
**Fl**	15	0.13	0.06	0.22	0–0.38	0.39	0.00	1.00	1.4–0.996	0.32	0.22	0.61	0–2.08
**P**	100	2.95	2.84	0.50	2.60–3.52	2.71	2.62	0.87	0–4.13	2.38	2.09	0.89	1.30–4.89
**A**	17	0.01	0.01	0.02	0–0.4	0.03	0.00	0.13	0–1.12	0.04	0.01	0.10	0–0.39
**Flu**	22	0.09	0.04	0.15	0–0.26	0.10	0.00	0.26	0–0.84	0.35	0.21	0.82	0–3.60
**Py**	17	0.09	0.05	0.16	0–0.28	0.34	0.00	1.03	0–4.17	0.30	0.02	0.79	0–3.80
**Ch**	60	0.57	0.60	0.54	0–0.63	0.52	0.30	0.94	0–4.07	0.73	0.16	2.62	0–14.51
**BghiPY**	8	0.06	0.03	0.11	0–0.2	0.05	0.00	0.17	0–0.71	0.29	0.23	1.01	0–5.16
**ΣPAHs**		**13.20**	**13.09**	**3.47**	**12.23–28.74**	**13.54**	**11.98**	**11.30**	**1.4–41.89**	**13.66**	**11.95**	**13.82**	**1.30–63.03**
**Carcinogenic PAHs**													
**BaA**	27	0.08	0.04	0.14	0–0.25	0.31	0.00	0.94	0–4.03	0.24	0.00	0.87	0–4.76
**B(b)Flu**	12	0.06	0.03	0.10	0–0.17	0.04	0.00	0.15	0–0.64	0.25	0.11	1.06	0–5.79
**B(k)Flu**	60	0.50	0.49	0.26	0.25–0.77	0.36	0.08	0.96	0–4.13	0.55	0.16	1.88	0–10.33
**BaPy**	41	0.41	0.44	0.39	0–0.47	0.49	0.39	0.95	0–4.18	0.46	0.14	0.98	0–4.12
**D(ah)A**	12	0.08	0.04	0.14	0–0.25	0.26	0.00	0.94	0–3.99	0.28	0.00	1.31	0–7.17
**IPy**	20	0.06	0.03	0.11	0–0.19	0.20	0.10	0.55	0–0.71	0.21	0.00	1.03	0–5.64
**ΣPAHs**		**1.20**	**1.07**	**1.14**	**0.25–2.1**	**1.67**	**0.57**	**4.48**	**0–17.65**	**1.99**	**0.41**	**7.13**	**0–37.81**

**Table 3:** PAHs concentrations (ng/mL) in human blood serum of COPD patients.

Compound (ng/mL)	(*n* = 51)	Former Smokers				Smokers				Non-Smokers			
COPD Group	Frequency (%)	Mean	Median	Stdev	Range	Mean	Median	Stdev	Range	Mean	Median	Stdev	Range
**Non-Carcinogenic PAHs**													
**N**	90	7.27	3.83	8.42	0–37.90	3.78	1.93	4.42	0.8–17.15	8.54	7.57	7.49	0.31–18.93
**Ace**	13	0.38	0.43	0.33	0.28–1.70	0.31	0.28	0.10	0.28–0.95	0.35	0.28	0.22	0.28–0.35
**Fl**	15	0.49	1.64	0.63	0.16–2.37	0.35	0.16	0.44	0.05–1.66	0.32	0.16	0.39	0.16–0.32
**P**	100	2.79	1.56	1.46	0.90–8.41	2.41	2.27	0.87	1.01–5.26	2.65	2.70	1.22	1.35–2.65
**A**	20	0.34	0.45	0.70	0.07–4.04	0.50	0.21	0.99	0.05–4.05	0.24	0.21	0.07	0.21–0.24
**Flu**	17	0.55	0.78	0.92	0.2–3.96	1.55	0.57	2.64	0.21–10.45	0.20	0.20	0.00	0.2–0.24
**Py**	43	1.00	0.76	1.78	0.16–9-9.18	1.97	1.09	2.81	0.00–11.19	0.82	0.26	1.04	0.26–0.82
**Ch**	59	0.75	0.85	1.38	0.04–6.96	1.18	0.41	1.76	0.13–6.37	1.32	0.17	3.33	0.02–1.32
**BghiPY**	17	0.70	0.68	1.36	0–6.83	0.82	0.29	1.24	0.29–5.11	2.04	0.29	5.25	0.29–2.04
**ΣPAHs**		**14.28**	**10.97**	**16.99**	**1.62–81.35**	**12.87**	**7.20**	**15.27**	**1.76–62.19**	**16.49**	**11.83**	**19.01**	**3.08–26.65**
**Carcinogenic PAHs**													
**BaA**	33	0.52	0.64	1.18	0–5.76	1.15	0.16	2.03	0.08–7.39	0.70	0.16	1.45	0.00–0.7
**B(b)Flu**	12	0.95	0.82	2.70	0.20–14.77	1.17	0.26	2.52	0.21–9.995	0.58	0.26	0.82	0.20–0.58
**B(k)Flu**	68	0.70	0.63	1.67	0.07–8.79	1.09	0.22	1.76	0.13–6.14	1.21	0.14	2.95	0.13–9.07
**BaPy**	67	0.56	0.65	0.90	0.09–3.99	1.21	0.35	2.02	0.09–7.38	0.87	0.21	2.08	0.09–6.41
**DahA**	24	0.74	0.72	1.20	0.02–5.98	1.16	0.68	1.99	0.00–8.18	1.79	0.36	4.04	0.36–8.55
**Ipy**	10	0.65	0.65	1.72	0–9.01	0.98	0.20	2.35	0.13–9.25	0.89	0.20	2.08	0.2–0.89
**ΣPAHs**	12	**4.12**	**4.11**	**9.36**	**0.38–48.3**	**6.76**	**1.88**	**12.65**	**0.63–48.29**	**6.04**	**1.33**	**13.43**	**0.98–26.2**

**Table 4:** Application of the Spearman correlation to contents of the PAHs in blood serum of smoker patients with COPD.

	N	Ace	Fl	P	A	Flu	Py	BaA	Ch	B(b)Flu	B(k)Flu	BaPy	D(a,h)A	BghiPy	IPy
N	1.00														
	-														
Ace	0.35	1.00													
	*p* ˂ 0.2	-													
Fl	0.91	0.47	1.00												
	**	*p* ˂ 0.08	-												
P	0.051	0.21	0.03	1.00											
	*p* ˂ 0.86	*p* ˂ 0.45	*p* ˂ 0.89	-											
A	0.19	0.08	0.12	0.53	1.00										
	*p* ˂ 0.51	*p* ˂ 0.78	*p* ˂ 0.67	*	-										
Flu	0.27	0.14	0.24	0.25	0.02	1.00									
	*p* ˂ 0.33	*p* ˂ 0.62	*p* ˂ 0.40	*p* ˂ 0.38	*p* ˂ 0.92	-									
Py	0.33	0.19	0.28	0.33	0.31	0.90	1.00								
	*p* ˂ 0.24	*p* ˂ 0.50	*p* ˂ 0.31	*p* ˂ 0.23	*p* ˂ 0.27	**	-								
BaA	0.31	0.13	0.22	0.44	0.49	0.83	0.95	1.00							
	*p* ˂ 271	*p* ˂ 0.64	*p* ˂ 0.44	*p* ˂ 0.11	*p* ˂ 0.72	**	**	-							
Ch	0.36	0.16	0.26	0.47	0.55	0.76	0.92	0.97	1.00						
	*p* ˂ 0.20	*p* ˂ 0.57	*p* ˂ 0.36	*p* ˂ 0.89	*	**	**	**	-						
B(b)Flu	0.24	0.10	0.17	0.21	0.01	0.98	0.93	0.87	0.81	1.00					
	*p* ˂ 0.40	*p* ˂ 0.73	*p* ˂ 0.55	*p* ˂ 0.45	*p* ˂ 0.96	**	**	**	**	-					
B(k)Flu	0.33	0.14	0.24	0.47	0.57	0.78	0.93	0.99	0.97	0.81	1.00				
	*p* ˂ 0.24	*p* ˂ 0.61	*p* ˂ 0.39	*p* ˂ 0.83	*	**	**	**	**	**	-				
BaPy	0.33	0.15	0.24	0.47	0.51	0.83	0.95	0.99	0.98	0.86	0.99	1.00			
	*p* ˂ 0.24	*p* ˂ 0.60	*p* ˂ 0.39	*p* ˂ 0.88	*p* ˂ 0.60	**	**	**	**	**	**	-			
D(a,h)A	0.26	0.16	0.18	0.27	0.01	0.95	0.91	0.83	0.79	0.97	0.78	0.83	1.00		
	*p* ˂ 0.35	*p* ˂ 0.58	*p* ˂ 0.52	*p* ˂ 0.33	*p* ˂ 0.97	**	**	**	**	**	**	**	-		
BghiPy	0.26	0.11	0.20	0.21	0.05	0.98	0.93	0.86	0.79	0.99	0.80	0.85	0.97	1.00	
	*p* ˂ 0.36	*p* ˂ 0.68	*p* ˂ 0.48	*p* ˂ 0.46	*p* ˂ 0.98	**	**	**	**	**	**	**	**	-	
IPy	0.22	0.09	0.15	0.22	0.02	0.97	0.92	0.86	0.81	0.99	0.80	0.86	0.96	0.98	1.00
	*p* ˂ 0.43	*p* ˂ 0.75	*p* ˂ 0.58	*p* ˂ 0.43	*p* ˂ 0.92	**	**	**	**	**	**	**	**	**	-

* *p* < 0.05, ** *p* < 0.005.

**Table 5:** Application of the Spearman correlation to contents of the PAHs in blood serum of non-smokers patients with COPD.

	N	Ace	Fl	P	A	Py	BaA	Ch	B(b)Flu	B(k)Flu	BaPy	D(a,h)A	BghiPy	IPy
N	1.00													
	-													
Ace	0.04	1.00												
	*p* ˂ 0.91	-												
Fl	0.28	0.15	1.00											
	*p* ˂ 0.46	*p* ˂ 0.68	-											
P	0.02	0.24	0.24	1.00										
	*p* ˂ 0.95	*p* ˂ 0.53	*p* ˂ 0.53	-										
A	0.35	0.13	0.14	0.10	1.00									
Py	0.62	0.20	0.51	0.13	-	1.00								
	*p* ˂ 0.07	*p* ˂ 0.60	*p* ˂ 0.15	*p* ˂ 0.73	*p* ˂ 0.58	-								
BaA	0.07	0.99	0.22	0.22	0.15	0.13	1.00							
	*p* ˂ 0.84	**	*p* ˂ 0.56	*p* ˂ 0.56	*p* ˂ 0.69	*p* ˂ 0.72	-							
Ch	0.04	0.99	0.16	0.23	0.14	0.20	0.98	1.00						
	*p* ˂ 0.90	**	*p* ˂ 0.66	*p* ˂ 0.54	*p* ˂ 0.707	*p* ˂ 0.59	**	-						
B(b)Flu	0.02	0.98	0.19	0.16	0.16	0.23	0.98	0.98	1.00					
	*p* ˂ 0.95	**	*p* ˂ 0.62	*p* ˂ 0.67	*p* ˂ 0.67	*p* ˂ 0.53	**	**	-					
B(k)Flu	0.03	0.99	0.16	0.21	0.15	0.21	0.98	0.99	0.98	1.00				
	*p* ˂ 0.93	**	*p* ˂ 0.67	*p* ˂ 0.57	*p* ˂ 0.69	*p* ˂ 0.58	**	**	**	-				
BaPy	0.02	0.99	0.14	0.23	0.15	0.20	0.98	0.99	0.99	0.99	1.00			
	*p* ˂ 0.94	**	*p* ˂ 0.71	*p* ˂ 0.54	*p* ˂ 0.69	*p* ˂ 0.59	**	**	**	**	-			
D(a,h)A	0.02	0.99	0.15	0.28	0.14	0.20	0.98	0.99	0.98	0.99	0.99	1.00		
	*p* ˂ 0.95	**	*p* ˂ 0.69	*p* ˂ 0.45	*p* ˂ 0.71	*p* ˂ 0.59	**	**	**	**	**	-		
BghiPy	0.04	1.00	0.15	0.24	0.13	0.20	0.99	0.99	0.98	0.99	0.99	0.99	1.00	
	*p* ˂ 0.91	*p* ˂ 0.93	*p* ˂ 0.68	*p* ˂ 0.53	*p* ˂ 0.72	*p* ˂ 0.60	**	**	**	**	**	**	**	
IPy	0.04	1.00	0.15	0.24	0.13	0.20	0.99	0.99	0.98	0.99	0.99	0.99	0.98	1.00
	*p* ˂ 0.91	*p* ˂ 0.67	*p* ˂ 0.68	*p* ˂ 0.53	*p* ˂ 0.72	*p* ˂ 0.60	**	**	**	**	**	**	**	-

** *p* < 0.005.

**Table 6:** Biochemical characteristics of the smokers/non-smokers/former smokers diagnosed with COPD.

		Mean	Median	Stdev	Range	Mean	Median	Stdev	Range	Mean	Median	Stdev	Range
Parameters	Lot	Ex-Smokers				Smokers				No-Smokers			
	Control	125.90	137.90	33.93	87.6–152.2	104.97	95.84	46.03	42.27–208.10	127.31	115.97	53.58	59.17–262.46
Triglycerides	COPD	103.09	88.00	63.87	10.76–267.60	226.99	94.07	422.78	42.22–1680.00	111.91	115.20	47.21	42.22–195.70
	Control	178.87	169.00	23.04	162.40–205.20	182.18	169.78	57.11	108.85–350.70	189.83	184.94	44.99	105.19–302.21
Cholesterol	COPD	187.01	188.00	42.84	110.20–268.60	184.33	155.42	94.20	120.00–488.14	175.23	180.00	49.69	107.3–269.40
	Control	56.96	45.75	35.00	28.94–96.19	41.68	42.00	15.12	21.79–81.12	41.61	41.67	13.05	17.39–76.98
HDL-C	COPD	45.37	44.92	18.80	20.73–81.12	44.93	47.26	14.11	14.25–66.00	48.08	46.55	12.00	25.58–66.00
	Control	91.14	88.08	39.36	53.39–131.94	113.08	109.94	50.40	50.88–277.17	117.11	118.69	42.62	41.14–221.27
LDL-C	COPD	103.93	102.00	29.03	60.15–183.26	87.82	81.04	33.12	48.49–161.14	90.14	92.70	24.74	59.13–133.16
	Control	2.41	2.25	0.50	2.01–2.97	2.35	2.44	0.62	1.20–3.24	2.50	2.44	0.43	1.72–3.44
MDA	COPD	2.43	2.30	0.99	0.70–6.92	2.72	2.37	1.15	1.81–5.55	2.32	2.41	0.60	1.43–3.18
	Control	4.90	5.10	0.82	4.00–5.60	4.90	4.67	1.61	2.03–9.20	5.72	5.60	2.55	2.15–16.70
Uric acid	COPD	6.09	5.60	1.45	3.09–9.23	5.21	5.22	0.86	3.51–6.84	6.50	7.10	1.60	2.61–7.70

**Table 7:** Mann–Whitney U test applied to contents of PAHs and biochemical parameters in the case of the COPD group.

	Rank Sum—COPD Std II	Rank Sum—COPD Std IV	U	Z	*p*-Value	Z-Adjusted	*p*-Value	Valid N—COPD Stage II	Valid N—COPD Stage IV
P	224.50	181.50	45.50	2.32	<0.02	2.32	<0.02	12.00	16.00
BaA	218.50	187.50	51.50	2.04	<0.04	2.22	<0.03	12.00	16.00
BaPy	221.00	185.00	49.00	2.16	<0.03	2.17	<0.03	12.00	16.00
UA	241.00	165.00	29.00	3.09	<0.00	3.10	<0.00	12.00	16.00

## Data Availability

Not applicable.

## Abbreviations

**Abbreviation**	**Compounds name**
N	Naphthalene
Ace	Acenaphtene
Acy	Acenaphthylene
Fl	Fluorene
P	Phenanthrene
A	Anthracene
Flu	Fluoranthene
Py	Pyrene
BaA	Benzo(a)anthracene
B(b)Flu	Benzo(b)fluoranthrene
B(k)Flu	Benzo(k)fluoranthrene
BaPy	Benzo(a)pyrene
DahA	Dibenzo(a,h)anthracene
BghiPy	Benzo(g,h,i)perylene
Ch	Chrysene
IPy	Indeno1,2,3-cd-pyren
